# Progress of single-cell sequencing technology in immunotherapy for tuberculosis

**DOI:** 10.3389/fcimb.2025.1666630

**Published:** 2025-09-22

**Authors:** Xinxin Fan, Muxing Chen, Di Wu, Youfei Lin, Xiaohong Chen

**Affiliations:** Department of Tuberculosis, Fuzhou Pulmonary Hospital of Fujian, Fuzhou, China

**Keywords:** single-cell sequencing, tuberculosis, immunotherapy, review, progress, precision medicine, host-directed therapy

## Abstract

According to the 2024 World Health Organization (WHO)Global Tuberculosis (TB)Report, tuberculosis remains the leading cause of death from a single infectious agent, with 10.8 million new cases and 1.25 million deaths in 2023. Early and standardized treatment upon definitive diagnosis holds significant importance for the prevention and prognosis of pulmonary tuberculosis patients. However, the number of drug-resistant tuberculosis(DR-TB) cases is increasing, while the interventions for tuberculosis are becoming increasingly limited. There is an urgent need to develop new rapid diagnostic methods and effective treatment drugs. Recent advances in tuberculosis immunotherapy have shown promising results. Novel therapeutic vaccines like M72/AS01E demonstrate 54% efficacy in preventing pulmonary TB, while host-directed therapies including nano-based drug delivery systems offer enhanced treatment outcomes. The immune system plays a vital role in the development and regulation of tuberculosis. Single-cell sequencing(SCS) technology enables comprehensive analysis of immune cells at the single-cell level, revealing the functions, states, distributions, and communication behaviors among immune cell subpopulations. These insights contribute to understanding the pathogenesis and discovering new diagnostic markers and therapeutic targets in tuberculosis. This review provides a critical overview of the immunological mechanisms underlying tuberculosis, immunotherapy for tuberculosis, and single-cell sequencing technology, with specific focus on key findings from recent studies and their clinical implications. It primarily focuses on discussing the research progress of single-cell sequencing technology in the context of tuberculosis immunotherapy and identifies current challenges and future research priorities.

## 1 Introduction

Tuberculosis, caused by Mycobacterium tuberculosis(MDR-TBMtb), is an infectious chronic disease that has plagued humanity for thousands of years (Furin et al., 2019). According to the 2024 WHO Global Tuberculosis Report, there were an estimated 10.8 million (95% CI: 10.1–11.7 million) new cases of tuberculosis worldwide in 2023, a small increase from 10.7 million in 2022 although still much higher than 10.4 million in 2021 and 10.1 million in 2020. The report highlights that drug-resistant TB continues to be a public health crisis, with approximately 400,000(95% UI: 360 000–440 000) new cases of Multidrug-Resistant Tuberculosis (MDR-TB) or rifampicin-resistant TB in 2023 (Organization W H, 2024). Undoubtedly, tuberculosis remains a major global health problem. Therefore, early diagnosis and treatment to curb the spread of tuberculosis are urgently needed. Recent advances in tuberculosis immunotherapy have opened new avenues for treatment. The M72/AS01E vaccine has shown 54% efficacy in preventing pulmonary TB in latently infected adults (Van Der Meeren et al., 2018). Cytokine-based therapies, including Interleukin-2 (IL-2) and Interferon-gamma (IFN-γ) supplementation, have demonstrated improved outcomes in MDR-TB patients (Grahmann and Braun, 2008; Tan et al., 2017). Additionally, nanotechnology-based drug delivery systems—such as liposomal formulations and polymeric nanoparticles—enable targeted, sustained, and specific delivery and may enhance TB therapy (Nair et al., 2023). Effective host immune responses can prevent the progression of tuberculosis in most cases, although some individuals may develop latent tuberculosis infection(LTBI). When the host immune function is compromised, latent tuberculosis may progress to active tuberculosis. Poor adherence to treatment regimens, improper use of anti-tuberculosis drugs, incorrect prescriptions by physicians, or poor drug quality can contribute to the development of drug-resistant tuberculosis. Some studies have found that in high-prevalence tuberculosis environments, there is still a small subset of individuals who possess innate resistance to developing latent Mycobacterium tuberculosis infection upon exposure (Stein et al., 2018). Therefore, it is important to gain a deeper understanding of host immune defense mechanisms against Mycobacterium tuberculosis and the current progress in tuberculosis immunotherapy. Single-cell sequencing allows for comprehensive analysis of immune cells at the single-cell level, and this technology can provide valuable information for tuberculosis diagnosis and precision treatment. This review aims to summarize the research progress of single-cell sequencing in tuberculosis immunotherapy, critically assess current limitations and methodological challenges, and propose future research priorities for clinical translation.

## 2 Immunological pathogenesis of tuberculosis

The host’s immune recognition, response, and regulation of Mtb determine the occurrence, development, and outcome of tuberculosis. Upon infecting the host, Mtb induces a series of innate and adaptive immune responses,while employing mechanisms to evade immune surveillance (Kilinç et al., 2021).

### 2.1 Innate immune response

The first line of defense is composed of airway epithelial cells (AEC), neutrophils (N), monocytes (M), and dendritic cells (DC), which rapidly to eliminate Mtb (Carabalí-Isajar et al., 2023).

In macrophages (differentiated from monocytes), Mtb arrests phagosome maturation to evade lysosomal degradation (Podinovskaia et al., 2013). This escape leads to dissemination in the lungs and extrapulmonary sites (Arora et al., 2017).

### 2.2 Adaptive immune response

CD4+ T cells producing IFN-γ are essential for protective immunity against Mtb (Cooper et al., 1993; Flynn et al., 1993; Urdahl et al., 2011; Cavalcanti et al., 2012). The programmed cell death protein 1 (PD-1)/programmed cell death ligand 1 (PD-L1) signaling pathway regulates T cell exhaustion, and PD-1 inhibition can restore Mtb-specific T cell function (Singh et al., 2014; Rai et al., 2016; Hu et al., 2020).

### 2.3 Cytokine balance

The balance between pro-inflammatory cytokines (interleukin-1 beta [IL-1β], IL-2, interleukin-12 [IL-12], IFN-γ, tumor necrosis factor-alpha [TNF-α]) and anti-inflammatory cytokines (interleukin-4 [IL-4], interleukin-13 [IL-13], interleukin-10 [IL-10]) significantly influences tuberculosis outcomes (Shaukat et al., 2023).

### 2.4 Granuloma formation

Granuloma are immune cell aggregates of tuberculosis (Ramakrishnan, 2012). While initially thought to benefit the host, recent studies reveal that granulomas can provide a niche for bacterial persistence and replication (Cronan et al., 2016; Pagán and Ramakrishnan, 2018). Single-cell sequencing has revealed significant heterogeneity in granuloma cellular composition, affecting treatment outcomes (Krausgruber et al., 2023; Qiu et al., 2024).

## 3 Tuberculosis immunotherapy

### 3.1 Current immunotherapeutic landscape

When the invasiveness of Mtb is imbalanced with the host immune response, individuals infected with Mtb may develop active tuberculosis. The disruption of this balance involves the dysregulation of multiple immunological mechanisms, including T-cell exhaustion, macrophage dysfunction, and the formation of an immunosuppressive microenvironment. Therefore, immunotherapy has great potential as a strategy for restoring and enhancing the host’s immune function. It is necessary to develop tuberculosis-specific immunotherapy drugs that can effectively regulate the anti-tuberculosis immune response, prevent and intervene in high-risk groups of tuberculosis infection or tuberculosis patients, and may provide new approaches for the combined treatment of tuberculosis and bring hope to patients with drug-resistant tuberculosis. According to the 2022 Expert Consensus on Immunotherapy for Tuberculosis, immunotherapeutic agents are classified as follows (Expert consensus on immunotherapy for tuberculosis (2022 edition), 2022):

1. Biological agents: Including therapeutic Vaccines (Mycobacterium vaccae (MV), Bacille Calmette-Guérin (BCG), Cytokine (IL-2,IFN-γ), and immunomodulators.
2. Host-directed therapy (HDT): Targeting host pathways to enhance immune responses.
3. Novel approaches: Including nano-based delivery systems, cell-based therapy, and checkpoint inhibitors.

### 3.2 Recent vaccine advances

Tuberculosis vaccines can be categorized into preventive and therapeutic vaccines. Preventive vaccines aim to avert Mycobacterium tuberculosis infection or arrest its progression to active tuberculosis disease, whereas therapeutic vaccination serves as an adjunctive treatment for individuals with LTBI and patients with active tuberculosis (ATB), while also preventing disease recurrence in those cured of tuberculosis. The BCG vaccine, derived from attenuated live strains of Mycobacterium bovis, is the first preventive vaccine used against tuberculosis. However, its efficacy varies among patients of different races, populations, and regions with varying tuberculosis incidence rates. The mechanisms underlying this variation remain incompletely elucidated. Evidence indicates that vaccine effectiveness is influenced by the characteristics of the infection source, vaccine-related attributes, and host-related determinants (Zimmermann and Curtis, 2019). BCG vaccination in neonates can provide protection for at least 20 years (Barreto et al., 2005), but it has minimal protective effects when administered to adults. The efficacy of BCG vaccination is poorest in children and elderly individuals with positive tuberculin skin test (TST) results (Mangtani et al., 2014). MV has shown significant efficacy and safety as an adjunctive therapy for multidrug-resistant tuberculosis (Weng et al., 2016), with higher rates of sputum conversion and radiographic improvement (Yang et al., 2011).

Internationally, various viral vector vaccines are under investigation, such as MVA85A (Tameris et al., 2013), the first novel TB vaccine in nearly 50 years to enter infant efficacy trials. Attenuated or recombinant live vaccines include MTBVAC (Spertini et al., 2015) and VPM1002 (Grode et al., 2005). MTBVAC, the only novel vaccine based on attenuated live Mtb, exhibits favorable safety and immunogenicity, inducing persistent antigen-specific T-helper 1 (Th1) cell responses in neonates (with stronger effects in high-dose groups compared to equivalent BCG doses) (Tameris et al., 2019). VPM1002, a recombinant BCG strain, demonstrates robust immunogenicity in adult trials (Grode et al., 2013) while being less reactogenic than BCG (Cotton et al., 2022).whole-cell or extract vaccines, such as RUTI and DAR-901 (Von Reyn et al., 2010), have shown promise. RUTI has proven effective in treating LTBI in animal models, reducing extrapulmonary Mtb dissemination. Recombinant protein/adjuvant vaccines like M72/AS01E provide at least three years of protection in Mtb-infected adults (Tait et al., 2019), with Phase IIb trials reporting 54% protective efficacy against TB disease (95% CI: 2.9%–78.2%) after two years, despite frequent adverse reactions including injection-site effects and flu-like symptoms (Van Der Meeren et al., 2018). MIP/Immuvac, as an adjunctive immunotherapy, may shorten the duration of treatment for tuberculosis (Chahar et al., 2018). Clinical trials of Tbvaccine have demonstratedefficacy in both prevention and treatment. However, most safety data derive from healthy adults, with insufficient evidence for populations with varying infection or immune statuses. Future vaccine designs should integrate cellular and humoral immunity mechanisms to address these gaps.

### 3.3 Host-directed therapy for tuberculosis

#### 3.3.1 Small molecule HDT drugs

Small molecule HDT drugs optimize anti-TB immunity by regulating host cell metabolism, autophagy and inflammatory responses. For instance, vitamin D can induce the generation of reactive oxygen species (ROS) and nitrogen intermediates, while inhibiting the expression of matrix metalloproteinases (MMP) such as MMP-9, MMP-10, and MMP-7 induced by Mtb infection in monocytes, thereby suppressing pro-inflammatory responses and reducing excessive tissue damage during active tuberculosis (Liu et al., 2007; Coussens et al., 2009). Metformin, on the other hand, enhances the production of mitochondrial ROS, stimulates the formation of phagolysosome-lysosome fusion, and restricts Mtb growth and replication (Singhal et al., 2014; Guler et al., 2021). The ROS-dependent neutrophil extracellular trap (NET) structure inhibitor LDC7559 holds therapeutic potential as it regulates excessive NET release while preserving the integrity of neutrophil host defense mechanisms (Sollberger et al., 2018). It should be noted that host-directed therapeutic drugs may have new therapeutic effects, but their side effects need to be considered. For example, metformin may decrease glycolytic efficiency in macrophages and acetyl-CoA production, potentially inducing ketosis (Viollet et al., 2012). Other immunomodulatory drugs such as nonsteroidal anti-inflammatory drugs (NSAIDs), like aspirin and ibuprofen, are candidate drugs for HDT, as they can enhance the anti-M. tuberculosis activity of pyrazinamide in mice infected with Mtb (Byrne et al., 2007). Critically, functional validation in TB models is essential to address heterogeneous responses.

#### 3.3.2 Mechanisms and challenges of cytokine therapy

HDT seeks to modulate the host’s inflammatory and cytokine responses, as well as autophagy—often using small molecules and biologics—to restrict mycobacterial infection. By acting on host pathways rather than directly on Mycobacterium tuberculosis (Mtb), HDT may reduce the likelihood of resistance (Roy et al., 2023). A surge of cytokine in drug-resistant tuberculosis is a hallmark of hyperinflammation and disease severity (Sampath et al., 2023), underscoring the central role of cell-mediated immunity in tuberculosis pathogenesis (Shaukat et al., 2023). Cytokine preparations, as core components of HDT, function by restoring or fine tuning host immune responses. For example, IL-2 can reverse T-cell exhaustion induced by chronic antigen stimulation (Liu et al., 2019), and low-dose IL-2 therapy shows potential to enhance immune responses in patients with MDR-TB (Johnson et al., 1997). Granulocyte-macrophage colony-stimulating factor (GM-CSF) inhibits Mtb growth in monocytes by promoting phagolysosomal fusion and autophagy; when combined with IL 2, it exhibits synergistic effects in animal models (Zhang et al., 2012). However, cytokine therapy faces notable challenges. First, its effects are dose and context dependent: although physiological levels of IFN γ are crucial for clearing Mtb (Dawson et al., 2009), overexpression can exacerbate tissue damage and immunopathology (Casanova et al., 2024). Second, many cytokines have short half lives and require frequent administration, increasing patient burden and treatment costs.

#### 3.3.3 Application of nanoscale drug delivery systems in anti tuberculosis treatment

Nanoscale drug delivery systems (NDDS) can enhance drug solubility, improve bioavailability, and increase tissue and cellular targeting, making them a promising modality in tuberculosis therapy (Garcia et al., 2022; Carnero Canales et al., 2024). A variety of nanocarriers have been explored, including nanoemulsions, liposomal nanocarriers, polymeric nanocarriers, gelatin-based nanocarriers, and inorganic nanocarriers (Buya et al., 2021). Multiple studies demonstrate that nano delivery systems enhance drug delivery to infected macrophages (Shen et al., 2023) and improve penetration into granulomas (Garcia-Contreras et al., 2021). They can directly target infection sites—such as the lungs (Wu et al., 2018), central nervous system (CNS) (Shobo et al., 2018), lymph nodes (Choudhary et al., 2022), and the skin (Hussain et al., 2020) and spine (Hikmawati et al., 2019)—to increase local drug concentrations, prolong drug release (Shah et al., 2020), enhance efficacy, reduce toxicity, and boost antibacterial activity against Mtb. Despite these advantages, NDDS still face limitations related to large scale manufacturing, biocompatibility and safety, overall cost effectiveness, and batch to batch reproducibility.

### 3.4 Other therapies

With the advancement of immunology and molecular biology, there is an increasing number of immunotherapeutic approaches being developed, including novel anti-tuberculosis drugs. Mesenchymal stem cells (MSCs), known for their strong regenerative and reparative abilities, have emerged as a novel modality for TB treatment. MSCs can phagocytose Mtb and restrict its growth through autophagy (Joshi et al., 2015). Patients receiving MSC therapy have shown a three-fold higher cure rate compared to those receiving only anti-tuberculosis treatment (Skrahin et al., 2016). Circulating Vγ9Vδ2 T lymphocytes represent a major innate peripheral T lymphocyte subset. immunotherapy using allogeneic Vγ9Vδ2 T cells has shown promise in reducing pulmonary lesions in TB patients, thus serving as a potential candidate for tuberculosis immunotherapy using cell-based therapeutics (Liang et al., 2021). However, further research is needed on issues such as the administration of large quantities of stem cells, route of administration, and timing. Manipulation of PD-1 signaling can restore host T cell responses, enhance protective immunity, and contribute to improved clearance of Mtb. Therefore, it has the potential to be used as an adjunctive immunotherapy for tuberculosis and serve as a biomarker to monitor host immune responses during treatment and vaccine research in tuberculosis patients (Singh et al., 2013). Rpf is a type of secreted protein produced by Mtb that stimulates mycobacterial growth and is associated with human TB infection (Mukamolova et al., 2010). Studies conducted by Romano and colleagues (Romano et al., 2012) found that Rv1009 (rpfB) and Rv2389c (rpfD) have the potential as plasmid DNA vaccines. Protein kinase G (PknG) is a virulence factor required for phagosome escape and contributes to the survival of non-replicating mycobacteria by promoting metabolic adaptation (Khan and Nandicoori, 2021). Arica-Sosa and colleagues (Arica-Sosa et al., 2022) discovered that RO9021 is a potential inhibitor of PknG.

HDT, particularly when combined with nano-delivery technology, introduces a revolutionary approach to treating TB. While immunomodulators show promise, their immediate efficacy in TB treatment cannot be guaranteed. Ongoing research is focused on developing a variety of novel immunomodulatory agents aimed at shortening treatment durations and improving cure rates. However, it is crucial to recognize that these agents can be a double-edged sword; improper application may lead to immunotolerance, compromising clinical outcomes. This underscores the importance of advanced techniques like single-cell sequencing, which are shaping our understanding of immune responses and enabling the development of more effective therapeutic strategies.

## 4 The application of single-cell sequencing technology in tuberculosis research

The emergence of single-cell genomics represents a turning point in cell biology. For the first time, we can determine the expression levels of each gene in the genome in thousands of individual cells within a single experiment. Sequencing is a core technology of genomics. Traditional second-generation sequencing technology typically provides the average transcriptome date of groups of cells, which can mask specific information among individual cells. As research progresses, it has become increasingly apparent that no two cells are identical, whether in structure or function. Therefore, traditional sequencing methods have failed to fully reveal the differences between cells. Since its advent in 2009, single-cell sequencing has become an important tool for studying the differences in cell populations and the evolutionary relationships of cells within organisms (Xue et al., 2015; Tang et al., 2019). Its core processes include the preparation of single-cell suspensions, single-cell isolation, nucleic acid amplification, high-throughput sequencing, and data analysis. Among these, single-cell isolation and nucleic acid amplification are crucial steps that directly affect the quality of subsequent data and the interpretation of results.

Single-cell sequencing technology can analyze the genomic, transcriptomic, and epigenomic maps of individual cells, revealing cellular heterogeneity, which is essential for understanding the pathogenesis of tuberculosis. However, obtaining high-quality single-cell samples from tuberculosis-infected tissues still faces many challenges. Firstly, during the sample preparation process, ensuring the activity, integrity, and low agglomeration rate of cells is vital. The processing methods for different types of samples vary, making it difficult to guarantee consistency in preparation. Additionally, technical variability may lead to batch effects, impacting data comparisons among different studies. Therefore, standardizing cross-laboratory protocols and processes is particularly necessary to ensure the comparability and reliability of data across different laboratories.

### 4.1 Preparation of single-cell suspension

The Preparation of single-cell suspension with high activity, good integrity, and low aggregation rate is crucial for the success of single-cell experiments. Different samples require different preparation methods. The basic steps for preparing a single-cell suspension from solid tissues include (Reichard and Asosingh, 2019) (1) Tissue dissociation: increasing the surface area of the initial solid tissue material to maximize contact between the tissue and digestion enzymes; (2) Enzymatic digestion: introducing an enzyme mixture into the chopped solid tissue to break down the extracellular matrix; and (3) cleaving cell-cell junctions. Peripheral blood mononuclear cells (PBMCs) in blood samples can be separated by density gradient centrifugation using Ficoll separation solution. It is worth noting that existing studies have shown that single-cell preparations of Mycobacterium tuberculosis may damage the bacterial envelope and interfere with macrophage interactions. Therefore, in studies of host-pathogen interactions, the influence of sample preparation methods must be carefully considered, as they may significantly alter the interpretation of bacterial mutants and have a significant impact on the Toll-like receptor 2 (TLR2)-dependent response in bone marrow-derived macrophages, as well as on the intracellular survival of bacteria (Mittal et al., 2023).

### 4.2 Single-cell isolation

Currently, several techniques are utilized for single-cell isolation (Gross et al., 2015), including mouth pipetting, serial dilution, laser capture microdissection (LCM), fluorescence-activated cell sorting (FACS) by flow cytometry, microfluidic chip technology, and microfluidics. Obtaining high-quality single cells from tissues infected with tuberculosis, especially granulomas, is a challenging task.

### 4.3 Nucleic acid amplification

Single-cell whole-genome amplification (scWGA) is an unbiased amplification method used to achieve high coverage and high fidelity amplification of the entire genome at the single-cell level.

Single-cell whole-transcriptome amplification (scWTA) is a technique that extracts RNA from isolated single cells, reversely transcribes captured mRNA into complementary DNA (cDNA), and amplifies the entire transcriptome using conventional polymerase chain reaction (PCR) or other *in vitro* transcription (IVT) methods (Kolodziejczyk Aleksandra et al., 2015).

### 4.4 High-throughput sequencing

High-throughput sequencing technology can simultaneously determine sequences of a large number of nucleic acid molecules in parallel. Generally, a single sequencing reaction can produce no less than 100Mb of sequencing data. Single-cell sequencing techniques primarily include single-cell DNA sequencing (scDNA-seq), single-cell RNA sequencing (scRNA-seq), single-cell epigenetic sequencing, single-cell proteomics analysis, single-cell spatial transcriptomics, and single-cell multi-omics integration analysis. High-throughput single-cell sequencing, with its extremely high-resolution, precisely analyzes the cellular composition information of samples, reveals the gene structure and expression status of individual cells on a large scale, and reflects the heterogeneity between cells and their relationships within the microenvironment.

#### 4.4.1 Single-cell DNA sequencing

Single-cell genome sequencing involves extracting DNA from a single cell, amplifying it, and performing high-throughput sequencing of the entire genome sequence of the target cell. This process enables the analysis of point mutations and copy number variations at the single-cell level, revealing differences between cells and their evolutionary relationships. This approach provides a new perspective for understanding host-pathogen interactions (Bertelli and Greub, 2013). Commonly used sequencing methods include ligation-mediated PCR (LA-PCR), probe enzyme protection-PCR (PEP-PCR), and degenerate oligonucleotide-primed PCR (DOP-PCR), among others.

#### 4.4.2 Single-cell RNA sequencing

Traditional transcriptomic sequencing techniques are based on population cells, and struggle to reflect the expression heterogeneity among individual cells. In contrast, single-cell transcriptome sequencing can more effectively assess cell diversity and identify new cell subtypes. Existing studies have shown that scRNA-seq has multiple applications in tuberculosis research, such as aiding in the understanding of the pathogenesis of tuberculous meningitis (Zhang et al., 2023) and elucidating the occurrence and potential mechanisms of T cell exhaustion in active Mtb infection (Pan et al., 2023).

#### 4.4.3 Single-cell epigenetic sequencing

Epigenetics can induce genetic phenotypic changes without altering the DNA sequence, including mechanisms such as DNA methylation and histone modification (Zhang et al., 2020). It reflects the gene-environment interaction in which genes determine traits (Dion et al., 2023). Through this technology, the influence of the environment on the host’s immune response can be explored (Chai et al., 2020).

#### 4.4.4 Single-cell proteomics analysis

By measuring the protein expression and modifications of cells, single-cell proteomics can provide important information about cell states and functions (O’donnell and Li, 2016). This method can analyze cell types and states even when the number of targets is limited (Shahi et al., 2017). Current methods include Protein Expression Analysis by Labeled Antibodies (PLAYR), transcriptomes and Epitopes by sequencing (CITE-seq), RNA Expression Analysis Pipeline (REAP-seq), Antibody sequencing (Abseq), among others. The conventional flow cytometry using fluorescently labeled antibodies has been widely used to sensitively analyze proteins in millions of single cells. SHAHI P and colleagues (Shahi et al., 2017) introduced a method called Abseq, which combines flow cytometry with mass spectrometry to analyze single-cell proteomics. This method utilizes specific antibodies to detect interested epitopes, and antibody-sequence tags are read through microfluidic barcoding and DNA sequencing. The Abseq method significantly improves sensitivity, accuracy, and multiplexing potential. Studies have shown that Proteasome Subunit Beta 9 (PSMB9), Signal Transducer and Activator of Transcription 1 (STAT1), and Antigen Processing 1 (TAP1) may play key roles in the pathogenesis of tuberculosis, and these genes and their protein products can also serve as diagnostic markers and potential therapeutic targets for the disease (Wu et al., 2023).

#### 4.4.5 Single-cell spatial transcriptomic sequencing

The dissociation of tissues and the isolation of cells disrupt the spatial information of cells within the native tissue environment. The cellular genome and transcriptome do not reflect the spatial distribution of each individual cell, whereas spatial transcriptomics sequencing can demonstrate the spatial expression patterns of genes. Spatial transcriptomics techniques enable the acquisition of transcriptomic data from intact tissue sections, thereby providing spatial distribution information and elucidating cellular interaction patterns (Longo et al., 2021). It serves as a powerful tool for studying the dynamics of complex structures, tissues, and organ systems, as well as the intrinsic mechanisms underlying their native contexts. Current methods include (Du et al., 2023) laser capture microdissection (LCM), single-molecule fluorescence *in situ* hybridization (smFISH), *in situ* sequencing (ISS), transcriptome *in vivo* analysis (TIVA), fluorescence *in situ* sequencing (FISSEQ), seqFISH, tomographic sequencing (tomo-seq), multiplexed error-robust fluorescence *in situ* hybridization (MERFFISH), single-molecule hydrogen bonding chain reaction (smHCR), Spatial transcriptomics, Geo-seq, NICHE-seq, BaristaSeq, ProximID, STARmap, osmFISH, slide-seq, seqFISH+, NanostringGeoMx DSP, DNA microscopy, APEX-seq, High Definition Spatial Transcriptomics (HDST), Zipseq, DBiT-seq, Exseq, Slide-seqV2, XYZeq, seq-Scope, sci-Space, stereo-seq, and Ex-ST, among others. Krausgruber T et al (Krausgruber et al., 2023). revealed the abnormal lymphoid development program driving granuloma formation through single-cell spatial transcriptome sequencing and established a comprehensive molecular and spatial landscape of non-infectious granulomas. This research provides a rich dataset for understanding the biological processes behind granuloma formation and lays the foundation for future explorations of therapeutic targeting.

#### 4.4.6 Single-cell multi-omics integration analysis

With the continuous development of single-cell sequencing (SCS) technology, researchers can now combine multi-omics sequencing to simultaneously obtain information on the single-cell genome, transcriptome, epigenetic modifications, proteome, and spatial transcriptome. This approach allows for a more complete understanding of cell information, including temporal and spatial expression. Integrated analysis can help us better understand cellular and molecular heterogeneity and their correlations in pathogenesis. Current methods include (Liu et al., 2023) Smart-RRBS, scMT-seq, scM&T-seq, scTrio-seq, scNMT-seq, iscCOOL-seq, scNOMeRe-seq, scGEM, epi-gSCAR, scNOMe-seq, scCOOL-seq, sci-CAR, NEAT-seq, SHARE-seq, among others. Recent studies have defined the complex multicellular ecosystem involved in granuloma regression and highlighted host immune targets that could be used to develop new vaccines and treatment strategies for tuberculosis (Gideon et al., 2022). Additionally, some studies have characterized specific subsets of lung macrophages that can limit or promote the growth of Mycobacterium tuberculosis (Pisu et al., 2021).

### 4.5 Data processing and analysis

To unravel the intertwined biological information and cope with the ever-increasing complexity of data, bioinformatics analysis is essential. With the rapid advancement of technology, various data analysis software, methods, and tools have been developed, encompassing sequence alignment, quality control, batch effect correction, dimensionality reduction, cell subtype identification, temporal and spatial analysis, among others. These approaches aim to maximize the advantages of single-cell sequencing technology and reduce the impact of current technological limitations. R and Python, as two widely used high-level programming languages, play a prominent role in the field of data science. Currently, data analysis in single-cell sequencing predominantly falls into two categories: extensive exploration of data through single-cell information and integration of multiple single-cell datasets (Hwang, 2023). Notable tools for single-cell sequencing analysis include (Liu et al., 2022) TopHat, SCnorm, Scran, Scater, Scanpy, Seurat, and M3Drop, Wishbone, Monocle, among others. Additionally, there are several widely-used databases for single-cell sequencing analysis (Liu et al., 2022), such as HTCA, TISCH, TEDD, ABC portal, Cancer SCEM, HCA, Jingle Bells, CancerSEA, scTPA, PanglaoDB, CellMarkrer, BloodSpot, scRNASeqDB, and Single Cell Portal. Furthermore, platforms for high-throughput single-cell sequencing technology include 10X Genomics Visium, Nanostring GeoMx DSP, Vizgen MERSCOPE, Illumina series, BGI-seq series, ABI SOLiD sequencer, Ion PGM, and others.

### 4.6 Advances in single-cell sequencing technologies

SCOPE-Seq (Yuan et al., 2018) is an scalable technique that combines live cell imaging with scRNA-seq to directly link phenotypic data, such as images, movies, or other cellular characteristics, to the whole transcriptome expression profile at the single-cell level. This technology does not require reverse transcription and amplification steps, but it does suffer from high sequencing error rates. Recently developed multimodal single-cell techniques, such as NEAT-seq, enable concurrent analysis of nuclear proteins, chromatin accessibility, and gene expression within individual cells. This multimodal approach enhances our ability to characterize cellular states and identify gene regulatory programs across diverse cell types (Chen et al., 2022). Consequently, we can now observe how nuclear changes propagate to the cell surface and vice versa for the first time (Pregizer et al., 2023). Asp M and colleagues (Asp et al., 2019) integrated spatial transcriptomics with scRNA-seq and employed *in situ* sequencing to localize cells within their original clusters, offering comprehensive insights into spatiotemporal patterns, marker genes, cell-cell interaction networks, and developmental trajectories. As single-cell technologies continue to advance, our understanding of individual cells deepens, and the integration of other techniques further enhances disease comprehension, target identification, and increases opportunities for disease cure.

## 5 The application of single-cell sequencing in tuberculosis immunotherapy

Tuberculosis has traditionally been classified into two major categories: LTBI and ATB. Clinical observations and research have revealed that hosts exhibit unique, complex, and individualized characteristics. Different hosts may share some common clinical manifestations of pulmonary tuberculosis, such as chronic cough, recurrent low -grade fever, night sweats, and fatigue;However the severity of these symptoms can vary significantly. It is worth noting that the risk of patients with latent tuberculosis infection progressing to active tuberculosis also shows significant individual differences. These observations highlight the heterogeneous manifestations and progression of the same disease in different hosts. Granuloma, as a key pathological diagnostic marker of tuberculosis, is characterized by a classic morphological structure: a caseous necrotic center surrounded by immune cells and fibroblasts. Research has found that granulomas in patients with active TB and LTBI exhibit morphological heterogeneity (O’garra et al., 2013). The formation of granulomas is a complex immune process that may either control and contain the infection (Russell et al., 2009) or promote the proliferation and spread of Mtb (Flynn, 2004). Differences in the composition of immune cells within granulomas may lead to completely different effects on Mtb—either promoting or inhibiting its growth. Variations in interaction patterns between Mtb and host immune cells may also result in different outcomes for granulomas. In addition, differences among Mtb strains may alter the progression trajectory of host granulomas, and the various states of granulomas may affect the activity and concentration of antibiotics. Therefore, the multi-level heterogeneity from the host to tissues and then to cells has a significant and unique impact on the occurrence and development of tuberculosis. With advancements in technology, fine analysis at the single-cell level has become possible. Single-cell sequencing technology has significantly enhanced the capacity for single-cell analysis (Wen and Tang, 2018). Through this technology, researchers can explore the heterogeneity, function, and interrelationships of tuberculosis immune cells, identify known, newly discovered, and rare tuberculosis pathogen targets, discover new therapeutic targets and disease biomarkers, characterize specific immune cell subsets in immunotherapy, and evaluate the impact of immunotherapy on cell subsets and cytokines in tuberculosis patients. This approach provides an in-depth understanding of drug sensitivity, drug resistance, and treatment modalities. Single-cell sequencing offers a more intuitive and comprehensive perspective, helping us understand tuberculosis immunotherapy and its latest developments.

Given that granulomas formed in zebrafish are extremely similar to those in humans, the zebrafish M. marinum model has become an effective system for studying the host and pathogen genetics of mycobacterial infection and granuloma formation (Davis et al., 2002). Cronan et al. (2021) found that atypical type 2 immune responses coordinated the formation and epithelialization processes of tuberculosis granulomas through scRNA-seq analysis of zebrafish granulomas and research on macaques infected with Mtb. High-throughput single-cell genomic analysis methods offer significant opportunities for defining the cell types, phenotypic states, and intercellular interactions that constitute granulomas, as well as for a deeper understanding of their dynamic changes (Prakadan et al., 2017). The Berit Carow team used ISS transcriptomics analysis and multiple immune markers on tissue sections to compare the immune microenvironment of granulomas in patients with tuberculosis and sarcoidosis. The study found that the formation of tertiary immune structures is a common feature of granulomas in tuberculosis patients, providing a new perspective for understanding the immune complexity of granulomas (Carow et al., 2023). Gideon et al. (2022) conducted multimodal analyses—including positron emission tomography-computed tomography (PET-CT) imaging, scRNA-seq, immunohistochemistry, and flow cytometry—on macaque lung granulomas, revealing cellular correlations related to tuberculosis control. Their research indicated that the cell ecosystem is enriched with type 1–17 and cytotoxic T cells, which are involved in pro-inflammatory signaling networks with multiple cell populations. In this cellular ecosystem, Mtb may be controlled or continue to survive and reproduce, providing new insights for developing host immune targets for tuberculosis vaccines and treatment strategies. The Winchell CG team determined through flow cytometry and single-cell RNA sequencing ligand-receptor analysis that interleukin-15 (IL-15) signaling is a key driver of cytotoxic T cells. Their research supports the idea that CD8(+) lymphocytes are crucial for the early control of Mtb infection, indicating that multifunctional cytotoxic responses may serve as potential vaccine targets (Winchell et al., 2023). This series of studies not only reveals the complex immune mechanisms underlying granuloma formation but also provides an important theoretical basis and potential strategies for precise immune interventions in tuberculosis.

With the further development of single-cell technology, these studies have provided key insights into single-cell immune responses. Single-cell immune profiling analysis enhances our understanding of responses at the single-cell level and facilitates the discovery of new diagnostic biomarkers and therapeutic targets. Single-cell RNA has shown that type I interferon (IFN-I)-responsive cells exhibit defects in their response to IFN-γ, which is crucial for controlling Mtb infection. Mrs. Koto’s research found that interstitial macrophages (IM) and plasmacytoid dendritic cells (pDC) are the primary producers of type I interferons, with pDC located near human Mtb granulomas (Kotov et al., 2023). Feng Y analyzed the characteristics of γδ T cell subsets in peripheral blood mononuclear cells (PBMCs), tuberculous pleural exudate (TPE), and pleural effusion from tuberculosis patients using single-cell sequencing. This study identified a subset of Vδ2 T cells with strong effector functions and high expression of FCGR3A, highlighting the functional diversity of γδ T cells in tuberculosis infection (Feng et al., 2025). The research by Villani et al. defined the heterogeneity of intermediate monocyte subpopulations through single-cell RNA sequencing indicating that these early identified subsets are highly heterogeneouss (Villani et al., 2017). Mtb can regulate the polarization of macrophage (Mily et al., 2020). By identifying the responses of different macrophage population to infection, we can better understand how immune cell response to Mtb infection. CHEN Q and colleagues performed single-cell sequencing on the alveolar lavage fluid of three patients with active pulmonary tuberculosis and found that alveolar macrophage subsets with increased proportions in these patients may initially polarize to M1 type and subsequently transform to M2 type. Additionally, there was a significant increase in the number and intensity of cell communications related to alveolar macrophages in pulmonary tuberculosis patients (Chen et al., 2022). SHARP J D and colleagues (Sharp et al., 2016) used chromatin immunoprecipitation sequencing (CHIP-Seq) to study the SigH binding sites that directly regulate the genes and operons of Mycobacterium tuberculosis, demonstrating the expansion of the SigH regulator and its regulatory response capabilities to various emergency situations. This work also underscores the significant value of a whole-genome approach in understanding the regulation of bacterial genes. It is noteworthy that immunomodulation-related genes are differentially expressed in tuberculosis patients. SONG J and colleagues (Song et al., 2023) established a competitive endogenous RNA(ceRNA) regulatory network using whole transcriptome sequencing and analyzed the regulatory non-coding RNAs involved in the pathological processes of tuberculosis. They identified has-miR-106a-5p, has-miR-17-5p, and has-miR-2355-5p as potential diagnostic biomarkers.Together, these studies provide new therapeutic targets for tuberculosis treatment and may drive innovations in treatment and prevention strategies.

Through single-cell sequencing technology, we can reveal the composition of T cell populations and immune cell subpopulations, thereby enhancing our understanding of their responses to Mtb infection. Wang Y and colleagues (Wang et al., 2023) utilized single-cell RNA transcriptome and T-cell/B-cell receptor (TCR/BCR) sequencing to unveil systemic immune dysregulation phenomena in patients with severe tuberculosis. CAI Y and colleagues (Cai et al., 2020) compared RNA-seq datasets from healthy controls (HC), individuals with LTBI, and TB patients, observing that natural killer (NK) cell subsets (CD3-CD7+GZMB+) were gradually depleted from HC to LTBI and TB stages. Their findings confirmed that changes in the frequency of this NK cell subgroup could effectively distinguish between TB patients, LTBI individuals, and HC. Additionally, Cai Y and colleagues (Cai et al., 2022) revealed the diversity of human T cell functions in tissues infected with tuberculosis and the specific amplification of T cell receptor (TCR) sequences within these tissues through single-cell sequencing technology. This knowledge about the composition and function of T cells at the infection site may assist in vaccine development. MUSVOSVI M and colleagues (Musvosvi et al., 2023) analyzed specific sequences of Mycobacterium tuberculosis in two longitudinal cohorts using single-cell and block TCR sequencing, alongside the GLIPH2 algorithm. This study provided a preliminary list of TCR specificities and established a comprehensive TCR sequence database, serving as a valuable tool for identifying candidate tuberculosis vaccine antigens. Finally, XU Y and colleagues (Xu et al., 2022) conducted a comprehensive analysis using both array and single-cell RNA-seq methodologies, finding that the expression of the immune-related hub gene ADM in peripheral blood could serve as a novel biomarker for differentiating tuberculosis from LTBI and HC. These findings provide promising ideas and insights for developing new treatment strategies for tuberculosis.

Through single-cell sequencing technology, we can gain a deeper understanding of the differences in gene expression among cells, thereby inferring the interactions within gene regulatory networks. This is particularly significant for understanding the potential responses of tuberculosis patients to immunotherapy. Kong et al. (2021) investigated the effects of secondary immune stimulation using bacterial lipopolysaccharide (LPS) and conducted single-cell transcriptomic sequencing before and after BCG vaccination. They found that BCG vaccination could reduce systemic inflammation and identified 75 genes that were affected by the LPS response, including inflammatory mediators with enhanced expressions, such as CCL3 and CCL4.This study elucidated the molecular mechanisms underlying BCG’s beneficial immune effects, highlighting the combination of a lower inflammatory state in circulation with an enhanced response of innate immune cells to reinfection. The changes associated with the training effects of BCG-induced circulating monocytes can offer valuable insights for the future enhancement of vaccine development.

Not all patients can benefit from current immunotherapy, and those who do respond to the treatment may develop drug resistance over time. Therefore, analyzing the mechanisms of drug resistance in immunotherapy is crucial for the effective application of these therapies and the formulation of individualized treatment strategies. Single-cell analysis holds promise for revealing the underlying causes of drug resistance and facilitating the development of tailored treatment plans. LEUNG K and colleagues (Leung et al., 2017) demonstrated that SMRT sequencing exhibited high stability in identifying clinical isolates of multidrug-resistant tuberculosis (MDR-TB). Through SMRT sequencing and comparative genomic analysis, researchers were able to identify mutations associated with the gradual development of drug resistance and the growth adaptability of MDR-TB in response to anti-tuberculosis drugs. The comparative genomic analysis also revealed new mutations at the rv0888, lpdA, and cobM gene loci, which may explain the differences in antibiotic resistance and growth patterns between the two MDR-TB resistant strains. Furthermore, Sehgal et al. (2021) utilized dynamic high-resolution scRNA-seq to investigate the fundamental mechanisms of immune escape in persistent anti-PD-1 responses within a functional model of immunotherapy. This approach provides an important reference for analyzing drug resistance in other treatment modalities or in conjunction with combination immunotherapy.

Immunotherapy offers hope to tuberculosis patients. The discovery of new targets and biomarkers is expected to shorten the treatment cycle for tuberculosis, enhance therapeutic efficacy, and reduce drug resistance. However, research on evaluating the effectiveness of tuberculosis immunotherapy through single-cell sequencing remains relatively scarce. As our understanding of the characteristics of immune cells, intercellular signal transduction, and changes in cytokines deepens, it will become increasingly possible to clarify the responses and prognoses of tuberculosis patients to immunotherapy in a more intuitive and effective manner.

## 6 Challenges and prospects

The heterogeneity of tuberculosis and the complexity of the host microenvironment lead to varying treatment responses and clinical outcomes. Advances in single-cell sequencing technology have provided an important tool for analyzing the heterogeneity of immune cells and the drug resistance associated with tuberculous meningitis (TBM). The application of this technology, coupled with the continuous development of spatial multi-omics platforms and comprehensive analysis of large-scale datasets, has significantly enhanced our understanding of tuberculosis heterogeneity and awareness of drug resistance. Furthermore, these advancements have contributed to innovations in diagnostic technologies for tuberculosis patients and the development of new drugs.

However, the application of single-cell technology faces several challenges and limitations. For instance, solid tissues must be processed into single-cell suspensions prior to sequencing, a complex and challenging procedure. Additionally, the binding activity of transcription factors cannot yet be measured at the single-cell level, and many fundamental statistical analysis issues, such as handling batch effects and technical variability, remain inadequately addressed (Trapnell, 2015). Although external RNA spike-in is the best method for quantifying technical noise, it has some limitations, such as the need to accurately calibrate the amount of external RNA spike-in added based on the total amount of endogenous RNA, and the inability to estimate the random loss of RNA molecules due to the effects of cell lysis efficiency (Kolodziejczyk Aleksandra et al., 2015). It is also noteworthy that some single-cell subtypes may not express major histocompatibility complex (MHC) Class II molecules in their resting state and can only be defined by non-RNA molecules or low-abundance transcripts, which may lead to these cells being overlooked in analyses (Villani et al., 2017). Moreover, technical issues related to RNA sequencing, such as library preparation, sequencing depth, and the influence of biological states, may result in data loss, as well as increased costs and the risk of numerous sequencing artifacts (Navin, 2014; Fittall and Van Loo, 2019). Standardizing protocols between different laboratories is key to addressing the current methodological consistency issues. Standardization can eliminate result variability caused by differences in experimental conditions, operational procedures, and data analysis methods. By establishing uniform experimental processes and standards, researchers can ensure the reproducibility and reliability of their results. Furthermore, this consistency can facilitate comparisons across studies, thereby promoting progress and collaboration in scientific research.

Effective drug targets identified *in vitro* need to be translated into effective therapeutic targets *in vivo* (Bellerose et al., 2020). Therefore, conducting *in vivo* analyses of drug targets is particularly important. By combining existing technologies, we should maximize the advantages of single-cell sequencing and integrate single-cell and spatial transcriptomics to gain a deeper understanding of the three-dimensional structure of granulomas. It is important to track immune responses during treatment to identify markers associated with drug resistance and develop targeted treatment strategies based on individual patient immune characteristics. Furthermore, optimizing the delivery mechanisms of nanomedicines through single-cell analysis is also an important objective. Overcoming these limitations and implementing precision medicine will provide new hope for tuberculosis patients.

In conclusion, single-cell sequencing technology has demonstrated significant potential in tuberculosis research, enabling us to gain a deeper understanding of the heterogeneity and drug resistance of immune cells involved in tuberculosis. With continuous advancements in this technology and the integration of spatial multi-omics platforms, we will be better equipped to analyze the complexity of tuberculosis and lay the groundwork for developing new diagnostic tools and therapeutic drugs. Despite this potential, challenges remain in the form of sample processing complexity, technical limitations, and high costs. Future research should focus on addressing these issues to achieve the goals of precision medicine and personalized treatment through technological innovation and interdisciplinary collaboration, ultimately providing more effective treatment options for tuberculosis patients and strengthening our overall response to this global health threat.

## Glossary

WHO: World Health Organization
TB: Tuberculosis
DR-TB: drug-resistant tuberculosis
SCS: Single-cell sequencing
Mtb: Mycobacterium tuberculosis
MDR-TB: Multidrug-Resistant Tuberculosis
IL-2: Interleukin-2v
IFN-γ: Interferon-gamma
LTBI: latent tuberculosis infectionv
AEC: airway epithelial cells
N: neutrophilsv
M: monocytesv
DC: dendritic cells
PD-1: programmed cell death protein 1
PD-L1: programmed cell death ligand 1
IL-1β: interleukin-1 beta
IL-12: interleukin-12
TNF-α: Tumor necrosis factor-alpha
IL-4: interleukin-4
IL-13: interleukin-13
IL-10: interleukin-10
ATB: active tuberculosis
BCG: Bacille Calmette-Guérin
TST: tuberculin skin test
MV: Mycobacterium vaccae
Th1: T-helper 1
HDT: Host-directed therapy
ROS: reactive oxygen species
NET: neutrophil extracellular trap
NSAIDs: nonsteroidal anti-inflammatory drugs
GM-CSF: Granulocyte-macrophage colony-stimulating factor
NDDS: Nanoscale drug delivery systems
CNS: central nervous system
MSCs: Mesenchymal stem cells
PknG: Protein kinase G
PBMCs: peripheral blood mononuclear cells
TLR2: Toll-like receptor 2
LCM: laser capture microdissection
FACS: fluorescence-activated cell sorting
scWGA: Single-cell whole-genome amplification
scWTA: Single-cell whole-transcriptome amplification
cDNA: captured mRNA into complementary DNA
PCR: polymerase chain reaction
IVT: *in vitro* transcription
HTS: High-Throughput Sequencing
scDNA-seq: single-cell DNA sequencing
scRNA-seq: single-cell RNA sequencing
LA-PCR: ligation-mediated PCR
PEP-PCR: probe enzyme protection-PCR
DOP-PCR: degenerate oligonucleotide-primed PCR
PLAYR: Protein Expression Analysis by Labeled Antibodies
CITE-seq: cellular indexing of transcriptomes and Epitopes by sequencing
REAP-seq: RNA Expression Analysis Pipeline
Abseq: Antibody sequencing
PSMB9: Proteasome Subunit Beta 9
STAT1: Signal Transducer and Activator of Transcription 1
TAP1: Transporter Associated with Antigen Processing 1
LCM: laser capture microdissection
smFISH: single-molecule fluorescence *in situ* hybridization
ISS: *in situ* sequencing
TIVA: transcriptome *in vivo* analysis
FISSEQ: fluorescence *in situ* sequencing
tomo-seq: tomographic sequencing
MERFFISH: multiplexed error-robust fluorescence *in situ* hybridization
smHCR: single-molecule hydrogen bonding chain reaction
HDST: High Definition Spatial Transcriptomics
PET-CT: positron emission tomography-computed tomography
IL-15: interleukin-15
IFN-I: type I interferon
IM: interstitial macrophages
pDC: plasmacytoid dendritic cells
PBMCs: peripheral blood mononuclear cells
TPE: tuberculous pleural exudate
ChIP-seq: chromatin immunoprecipitation sequencing
ceRNA: competitive endogenous RNA
TCR/BCR: T cell/B cell receptor
HC: healthy controls
NK: natural killer
LPS: lipopolysaccharide
TBM: tuberculous meningitis
MHC: major histocompatibility complex
